# Role of EDA Fibronectin and Toll-like Receptor 5 in the Development of the Tumor Microenvironment in Triple Negative Breast Cancer

**DOI:** 10.3390/cancers18162693

**Published:** 2026-08-20

**Authors:** Anthony Ambesi, Hailey Reed, Paula McKeown-Longo

**Affiliations:** Department of Regenerative & Cancer Cell Biology, Albany Medical College, Albany, NY 12208-3479, USA; ambesit@gmail.com (A.A.); hqreed@gmail.com (H.R.)

**Keywords:** triple negative breast cancer, cancer-associated fibroblasts, TLR5, EDA-fibronectin, TGF-β, tumor microenvironment, inflammatory cytokine

## Abstract

**Background/Objectives**: Triple negative breast cancer remains the most difficult breast cancer to treat as there are no specific targeted therapies. The tumor stroma is recognized as essential for the development of solid tumors. Cancer associated myofibroblasts are known to play a major role in the construction of a tumor microenvironment conducive to tumor progression. Therefore, we established a co-culture system to identify potential molecular targets controlling myofibroblast conversion. **Methods**: Co-cultures of MDA-MB-468 triple negative breast cancer cells and skin fibroblasts were either mixed prior to seeding or seeded individually on opposite sides of a culture dish and incubated in complete medium. Inhibitors were preincubated with cells for 1 h. Inflammatory cytokines were measured by ELISA. Proteins were analyzed using the Wes-Protein Simple System. Immunostaining was visualized using a Hamamatsu Photonics Nanozoomer. Statistical analysis was done by student’s *t*-test and one-way ANOVA. **Results**: The data indicate that co-culturing the cells promotes myofibroblast differentiation and cytokine release and this requires direct contact between the two cell types. Both myofibroblast conversion and cytokine release were prevented by inhibitors of TLR5, TGF-β and EDA-fibronectin. **Conclusions**: Data suggest that the TLR5 receptor on the MDA-MB-468 cells binds to EDA-fibronectin expressed by fibroblasts thus inducing inflammatory cytokine expression. Expression of EDA fibronectin is regulated through TGF-β whose synthesis is induced by TLR5 signaling in the tumor cells. The data are consistent with a model in which TLR5-mediated crosstalk between the cancer cells and the stromal fibroblasts creates a tissue microenvironment conducive to tumor growth and metastasis.

## Simple Summary

Triple negative breast cancer is the most difficult breast cancer to treat due to the lack of specific drug targets. We evaluated whether the tumor microenvironment might provide opportunities for the identification of novel targets for treatment. Using a co-culture system of MDA-MB-468 triple-negative cancer cells and human skin fibroblasts, we identified the interaction of tumor cell TLR5 with the EDA domain of fibronectin as essential to the conversion of fibroblasts to cancer-associated myofibroblasts and to the release of the pro-tumorigenic inflammatory cytokines, IL-6 and IL-8.

## 1. Introduction

Breast cancer remains the second leading cause of cancer death in women. Triple-negative breast cancer (TNBC) accounts for approximately 20% of all breast cancers, and therapeutics to specifically target TNBC are not yet available [1,2]. Stage 1 TNBC currently lacks adjuvant therapeutics [3]. It is now recognized that the tumor microenvironment plays a critical role in tumor progression and metastasis. Growth and progression of solid tumors, including TNBC, occur in a microenvironment characterized by fibrosis and inflammation, thus creating the tissue rigidity integral to tumor growth and metastasis [4]. The tumor environment consists of stromal fibroblasts, adipocytes, immune cells and extracellular matrix. Intercellular crosstalk between tumor cells and stromal fibroblasts results in the differentiation of fibroblasts into Cancer-Associated Fibroblasts (CAFs). CAFs are highly contractile myofibroblasts characterized by the increased expression of alpha-smooth muscle actin (α-SMA) [5]. CAFs are primarily responsible for the assembly and structural organization of the tumor microenvironment. The CAFs increase the synthesis and deposition of a stiffened extracellular stromal matrix enriched for the alternatively spliced EDA+ isoform of fibronectin (FnEDA) [6,7].

FnEDA has been identified as a Damage Associated Molecular Pattern molecule (DAMP) which activates Toll-like receptors (TLRs) to initiate innate immune responses in a variety of cell types [8,9,10,11]. TLRs represent a nine-member family of receptors that function as homo- or heterodimers to regulate transcription of fibro-inflammatory genes. The TLR receptors that recognize the EDA domain of fibronectin are cell-type specific. Fibroblasts bind the EDA domain of fibronectin using the TLR4 homodimer [12] while the MDA-MB-468 TNBC cells bind the EDA domain using the TLR5 homodimer [13]. TLR binding to FnEDA, either as the individual Type III domain or within the context of the complete protein, causes the activation of NFκB and MAP kinases to initiate transcription of fibro-inflammatory genes. Additionally, the EDA domain of fibronectin plays an important role in the activation of TGF-β by providing a binding site for TGF-β in the extracellular matrix where it is subsequently activated by the action of integrin receptors and metalloproteases [14].

In the current study, we have used a co-culture system of human stromal fibroblasts and MDA-MB-468 TNBC cells to identify the steps that regulate the conversion of fibroblasts to myofibroblasts to identify possible drug targets for the treatment of TNBC. Our findings indicate that the EDA domain of fibronectin and the TLR5 receptor play a critical role in the development of the TNBC microenvironment.

## 2. Materials and Methods

### 2.1. Antibodies and Reagents

The following antibodies were obtained from Cell Signaling Technology (Danvers, MA, USA): alpha-smooth muscle actin (αSMA; 19245), TGF-β (3709) and GAPDH (2118). Antibodies to vimentin (sc-5565) and fibronectin extra domain A (FnEDA; Ist-9, sc-59826) were obtained from Santa Cruz Biotechnology, Inc. (Santa Cruz, CA, USA). TLR5 neutralizing antibody (maba2-htlr5) was obtained from InvivoGen (San Diego, CA, USA), and the TLR5 inhibitor, TH1020, was from R&D Systems Inc. (Minneapolis, MN, USA). Doses of TH-1020 used were based on prior publications [15,16]. The polyclonal antibody directed against full-length human plasma fibronectin (total Fn) used in this study was previously described [17]. Purified recombinant fibronectin EDA domain (FnEDA) was prepared and purified as previously described [11]. Bexotegrast (PLN-74809; cat HY-137561) and irigenin (HY-N2587) were obtained from MedChem Express (Monmouth Junction, NJ, USA), and the IL-8 and IL-6 ELISA assay kits were obtained from BD Biosciences (San Diego, CA, USA). Recombinant human TGF-β1 (rhTGF-β1; 240-B) was obtained from R&D Systems Inc. (Minneapolis, MN, USA). SB-431542 (TGF-β1 receptor inhibitor; A8249) was obtained from Apex Biotechnology Corp. (Boston, MA, USA).

### 2.2. Cell Culture

Human dermal fibroblasts (A1F) were a gift from Dr. Lynn Allen-Hoffman at the University of Wisconsin [17,18]. Both the fibroblasts and the human triple negative breast cancer cells MDA-MB-468 (ATCC; HBT-132) were maintained in complete Dulbecco’s modified eagle medium (DMEM; Invitrogen/Life Technologies, Corp., Grand Island, NY, USA) supplemented with 1% Pen-Strep (Gibco, Waltham, MA, USA), 1% GlutaMAX (Gibco) and 10% fetal bovine serum (FBS; Hyclone Laboratories, Logan, UT, USA) in a humidified chamber at 37 °C/8% CO_2_.

### 2.3. Cell Treatments

Unless otherwise stated, cells were plated onto 24-well culture plates in complete medium for 3–4 days prior to collecting conditioned medium and cell lysates. Treatment with inhibitors (irigenin, bexotegrast or the anti-TLR5 neutralizing antibody) was typically carried out in complete medium for 1 h prior to addition of cells to coated dishes, addition of TGF-β or co-culture. Specific treatments are described in figure legends. IL-8 and IL-6 were measured using human enzyme-linked immunosorbent assay (ELISA) kits obtained from BD Biosciences (San Diego, CA, USA) and performed according to the manufacturer’s recommended procedure.

### 2.4. Protein Analysis Using the Wes-Protein Simple System

Whole-cell lysates were obtained by first rinsing cell layers twice with ice-cold phosphate-buffered saline (PBS) containing 1 mM sodium ortho-vanadate, followed by direct lysis in SDS-containing sample buffer (62.5 mM Tris, pH 6.8, 2% SDS, 10% glycerol, 50 mM dithiothreitol, and 0.01% bromophenol blue). Samples were denatured by heating at 95 °C for 6 min and an automated capillary-based western system (Wes-ProteinSimple, San Jose, CA, USA) was used for protein detection according to the manufacturer’s recommended procedure. Quantitative analysis of targeted proteins was performed with Compass system software 3.1.7 (ProteinSimple) and quantified relative to vimentin or GAPDH expression. Electropherograms obtained from the Wes-Protein Simple analysis are presented in Supplemental Figures S1–S8.

### 2.5. Fluorescence Microscopy

A1F fibroblasts and MDA-MB-468 breast cancer cells were cultured for up to 4 days on glass cover slips in complete medium. Cells were fixed for 20 min in 3% paraformaldehyde, permeabilized in 0.5% Triton X-100 for 10 min, blocked in 1% BSA, and immunostained for αSMA and fibronectin EDA domain (FnEDA) and visualized with Alexa Fluor-conjugated secondary antibodies. Nuclei were counterstained with DAPI. Coverslips were mounted onto glass slides and cell layers were examined on a Hamamatsu Photonics Nanozoomer (Hamamatsu Corporation; Bridgewater, NJ, USA).

### 2.6. Statistical Analysis

Quantitative results were reported as mean ± standard error of the mean (SEM). Student’s *t*-test was used to compare the levels of two experimental groups, and One-Way ANOVA was used for comparison over two groups. The significance level was set at 0.05, and SigmaPlot 12.5 (Systat Software, Chicago, IL, USA) was used for statistical analysis.

## 3. Results

**Figure 1:**
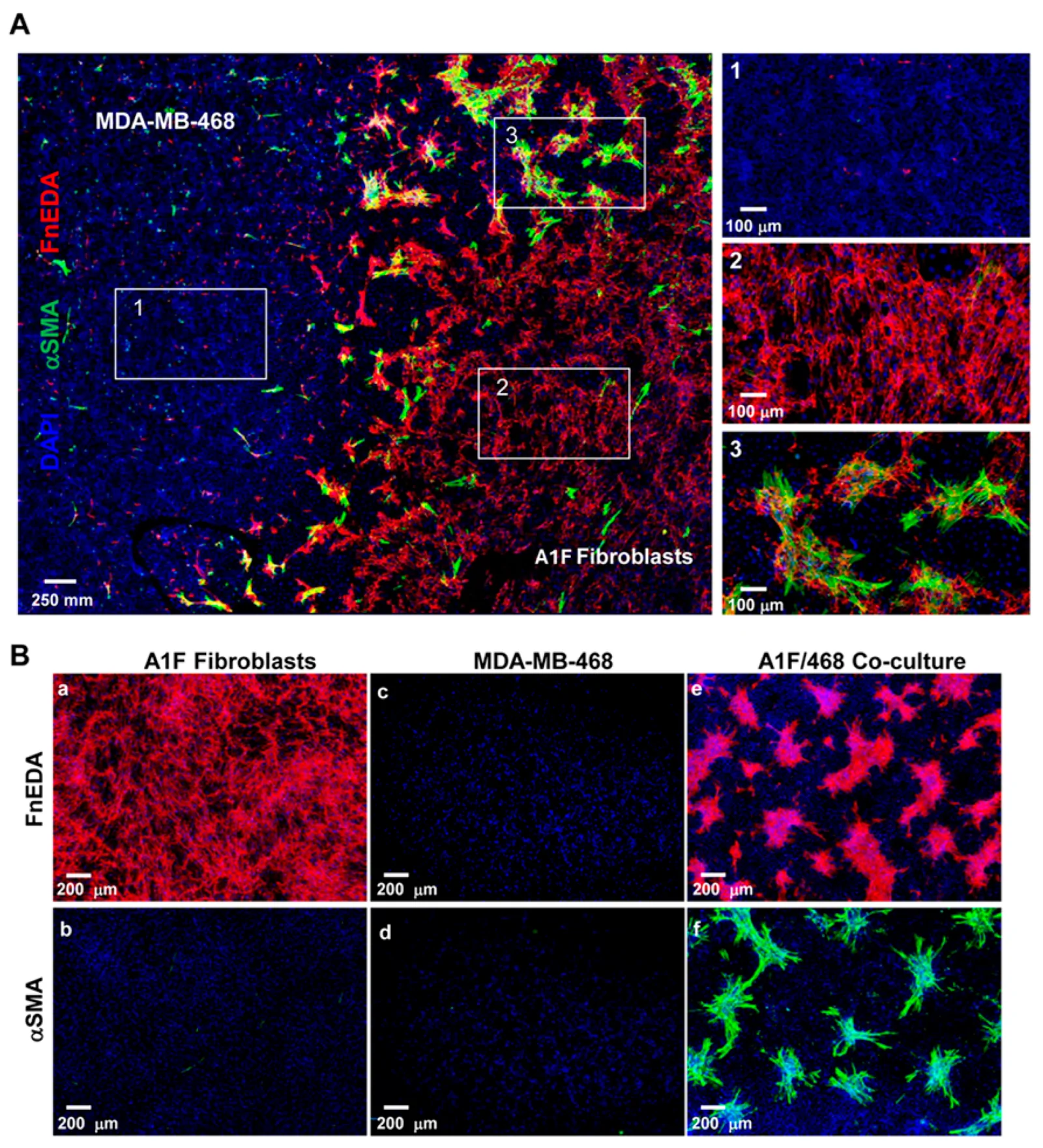
MDA-MB-468 triple-negative breast cancer cells induce expression of alpha smooth muscle actin and EDA-fibronectin in adjacent A1F dermal fibroblast cells. (**A**) MDA-MB-468 breast cancer cells (inset 1) and A1F fibroblasts (inset 2) were seeded separately onto opposite sides of a glass coverslip and allowed to proliferate for 4 days in complete medium. A convergence region of mixed-cell interaction is identified (inset 3). Cultures were rinsed, and cells were stained for alpha smooth muscle actin (αSMA) and EDA-fibronectin (FnEDA); nuclei were counterstained with DAPI. (**B**) A1F (**a**,**b**) and MDA-MB-468 (**c**,**d**) cells were cultured in complete medium separately or in co-culture (**e**,**f**). After 3 days, cells were fixed and stained for FnEDA (**a**,**c**,**e**) or αSMA, and nuclei were counterstained with DAPI (**b**,**d**,**f**).

**Figure 2:**
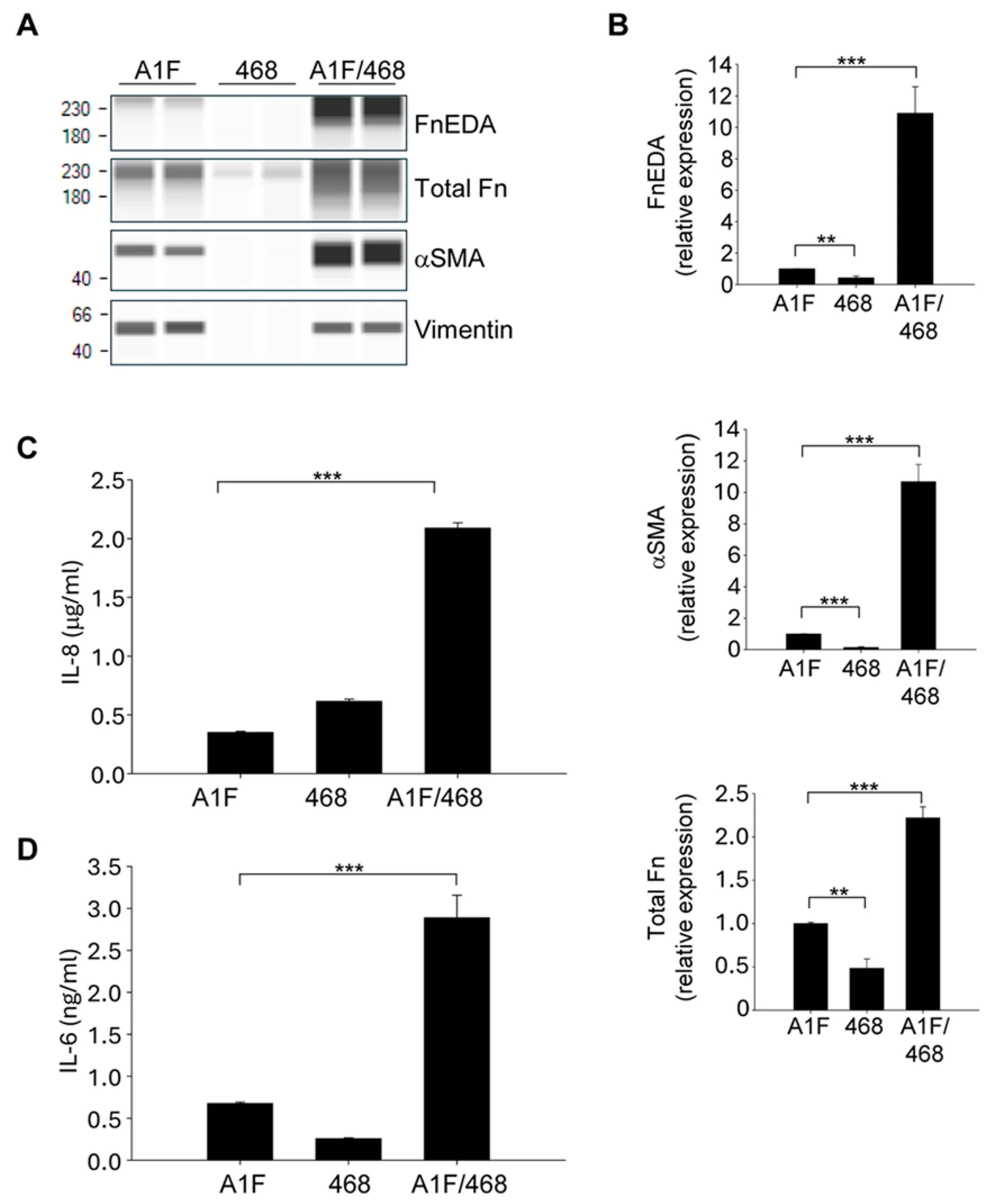
Mixed cultures of MDA-MB-468 breast cancer cells and A1F fibroblasts exhibit increased expression of fibro-inflammatory genes. A1F fibroblasts (A1F) and MDA-MB-468 breast cancer cells (468) were cultured either alone or in mixed culture (A1F/468) for 3 days (**A**). Cell lysates were analyzed using Simple-WES and probed for FnEDA, total fibronectin (total Fn) and αSMA. Vimentin served as loading control. Relative protein expression is quantified in (**B**). Conditioned medium was harvested and assayed by ELISA for IL-8 (**C**) and IL-6 (**D**). ** *p* < 0.01; *** *p* < 0.001.

To characterize the impact of cancer cells on the neighboring stromal fibroblasts, human triple-negative breast cancer cells, MDA-MB-468, were co-cultured with human skin A1F fibroblasts. Fibroblasts and cancer cells were seeded onto opposite sides of a tissue culture dish in complete medium and cultured for 6 days (Figure 1A). Cells were fixed and stained for FnEDA and αSMA. Nuclei were stained with DAPI (Figure 1A, panel 1). As expected, the fibroblasts show extensive staining (shown in red) for FnEDA in the extracellular matrix (Figure 1A, panel 2). However, the fibroblasts immediately adjacent to the MDA-MB-468 cells stain for both FnEDA as well as αSMA (shown in green), consistent with their differentiation into CAFs (Figure 1A, panel 3). The staining for αSMA was more intense in areas of closer cell contact (compare panels 1,2,3), suggesting that direct cell contact between the tumor and the fibroblast cells promotes the differentiation of fibroblasts into CAFs. The findings in Figure 1A were further supported by the data shown in Figure 1B. A1F cells (a,b) cultured separately from 468 breast cancer cells (panels c,d) were stained for either FnEDA (panels a,c,e) or αSMA (panels b,d,f). The fibroblasts did assemble an extensive FnEDA-containing extracellular matrix (panel a) but expressed only trace amounts of αSMA (panel b). MDA-MB-468 cells were negative for both FnEDA (panel c) and αSMA (panel d). Staining for αSMA is observed when the cancer cells are mixed with the fibroblasts prior to co-culture (panels e,f), consistent with the conversion of fibroblasts to CAFs. Additionally, the cells have assembled into clusters, indicating that the expression of αSMA is accompanied by an active increase in contractile forces [14]. These data were quantified in Figure 2. WES analysis (Figure 2A) indicates that mixing the fibroblasts and tumor cells results in an approximate 10-fold increase in the expression of both FnEDA and αSMA (Figure 2B), consistent with the conversion of fibroblasts to myofibroblasts. In addition, contact between the two cell types also increases the levels of IL-8 and IL6, cytokines known to promote triple-negative breast cancer tumor growth, invasion [19,20], and therapeutic resistance [21].

**Figure 3:**
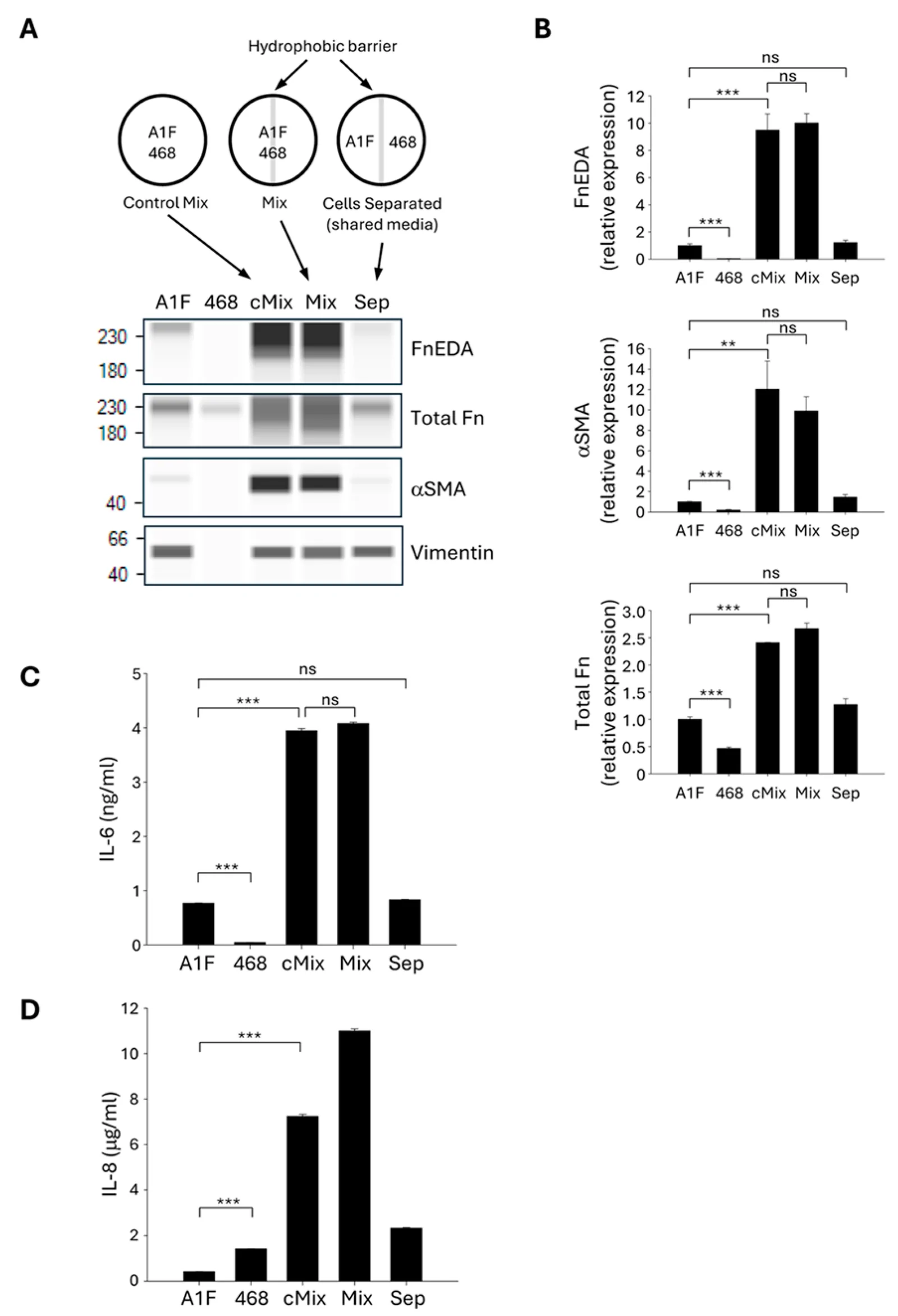
Enhanced expression of αSMA and FnEDA requires direct contact between MDA-MB-468 breast cancer cells and A1F fibroblasts. A grease marker was used to generate a hydrophobic barrier that physically separated (Sep) the adhesive culture area of a multi-well tissue culture plate, thereby preventing direct contact between cells cultured on either side of the barrier. MDA-MB-468 breast cancer cells and A1F fibroblasts were cultured in complete medium separately on opposing sides of the barrier (shared culture medium; no direct contact). Mixed cultures were seeded onto wells with a hydrophobic area (Mix). Cells seeded onto wells without the hydrophobic barrier served as positive controls (cMix). After 3 days in culture, cell lysates were analyzed by Simple-WES and probed for FnEDA, total Fn, and αSMA (**A**). Vimentin served as loading control. Relative expression is quantified in (**B**). Conditioned medium was harvested and assayed for IL-6 (**C**) and IL-8 (**D**). ** *p* < 0.01; *** *p* < 0.001; ns = not significant.

To determine whether the conversion of A1F fibroblasts to CAFs required direct contact with cancer cells, each cell type was seeded onto separate sides of a tissue culture dish. As depicted in Figure 3A, areas were separated using a hydrophobic barrier, which allowed for mixing of the culture medium while prohibiting contact between the two cell types. A1F and 468 cells, which were mixed prior to seeding, served as positive control. After 72 h of culture, cells were solubilized, and expression of αSMA and FnEDA was assessed by WES (Figure 3A). These data are quantified in Figure 3B, which shows that the mixed cultures (cMix, Mix) demonstrated approximately a 10-fold increase in both FnEDA and αSMA as well as a 2.5-fold increase in total fibronectin, suggesting that the 10-fold increase in FnEDA is primarily due to an increase in alternative splicing of the EDA exon. Cells that were separated by the hydrophobic barrier (Sep) did not show any increase in either FnEDA or αSMA. These data are consistent with myofibroblast conversion requiring direct contact between the cancer cells and the fibroblasts. In addition, the increase in IL-6 and IL-8 also required cell–cell contact (Figure 3C,D).

**Figure 4:**
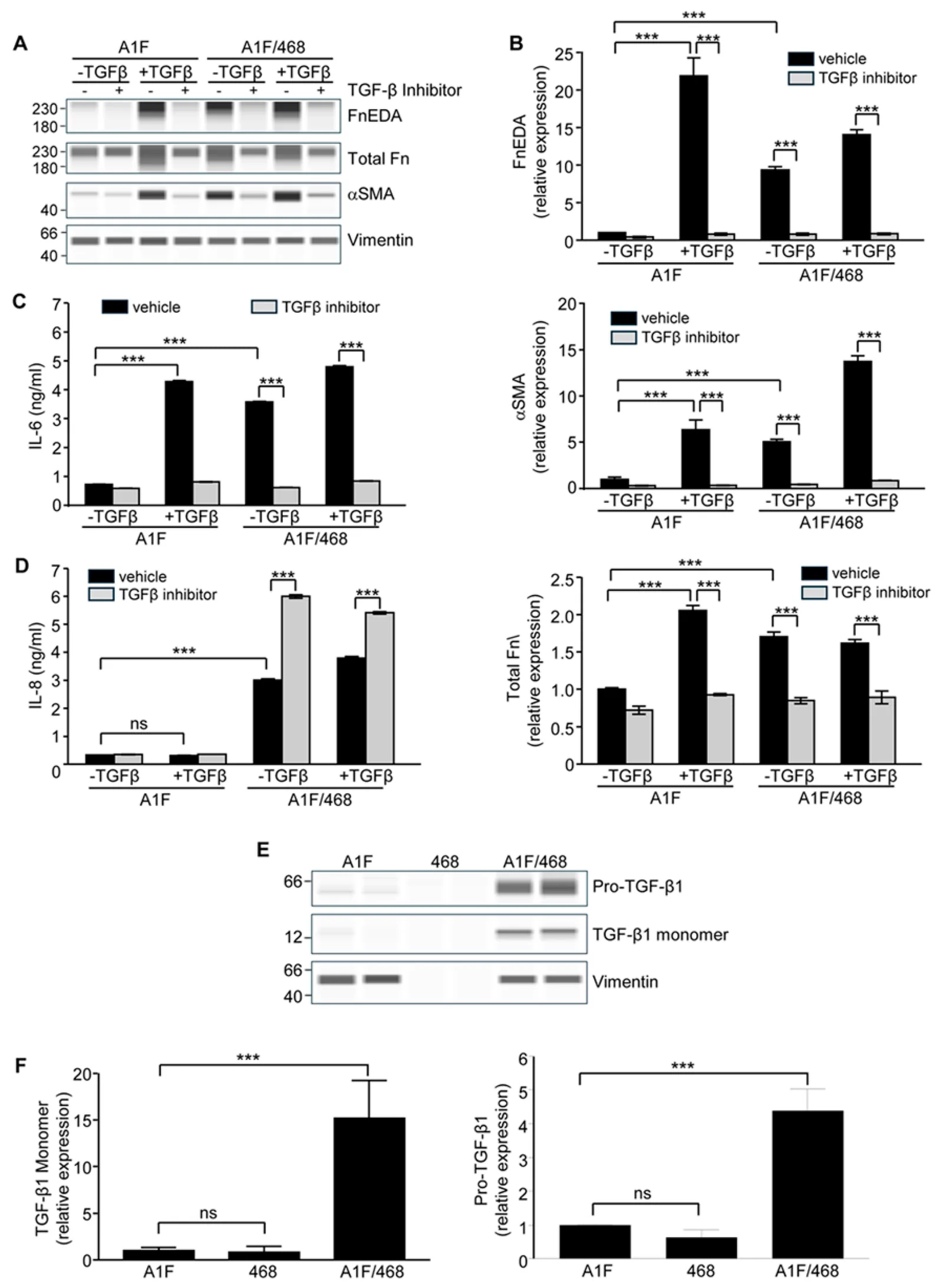
MDA-MB-468 breast cancer cell-induced myofibroblast conversion is TGF-β dependent. A1F fibroblasts were seeded alone (A1F) or in co-culture with MDA-MB-468 breast cancer cells (A1F/468) in complete medium and treated with or without the TGF-β receptor inhibitor, SB43152 (10 μM), for 1 h prior to the addition of TGF-β (5 ng/mL). Cells were then incubated for 3 days. After 3 days in culture, conditioned medium was harvested, and cell lysates were prepared. Lysates were subjected to Simple-WES and analyzed for FnEDA, total Fn and αSMA (**A**). Vimentin served as loading control. Relative expression of proteins is shown in (**B**). Conditioned medium was analyzed by ELISA for IL-6 (**C**) and IL-8 (**D**). A1F fibroblasts (A1F) and MDA-MB-468 breast cancer cells (468) were cultured separately or in co-culture(A1F/468) for 3 days in complete medium (**E**). Cell lysates subjected to Simple-WES were probed for Pro-TGF-β or TGF-β1 monomer (**E**) and relative expression determined (**F**). Vimentin served as loading control. *** *p* < 0.001; ns = not significant.

As FnEDA and αSMA are typically induced by TGF-β, the TGF-β receptor inhibitor (SB 431542) was tested for its effects on the expression of FnEDA and αSMA in mixed cell cultures. As shown in Figure 4A, the mixed cultures (A1F/468) induced expression of FnEDA and αSMA. Addition of the TGF-β receptor inhibitor, SB431542, to the mixed cultures prevented the induction of FnEDA and αSMA. Cultures of the A1F cells treated with TGF-β served as positive controls. These data are quantified in Figure 4B. The induction of IL-6 in the co-cultures was also inhibited by SB431542, indicating that IL-6 expression was dependent on TGF-β (Figure 4C). In contrast, the TGF-β inhibition resulted in an increase in IL-8 expression (Figure 4D). This observation is consistent with earlier studies showing that IL-8 is negatively regulated by TGF-β1 [22,23,24]. WES analysis of culture media shows a 4-fold increase in the expression of pro-TGF-β1 and a 15-fold increase in the active TGF-β monomer by the cocultures (Figure 4E,F), suggesting that cell–cell contact was required for the activation of TGF-β.

**Figure 5:**
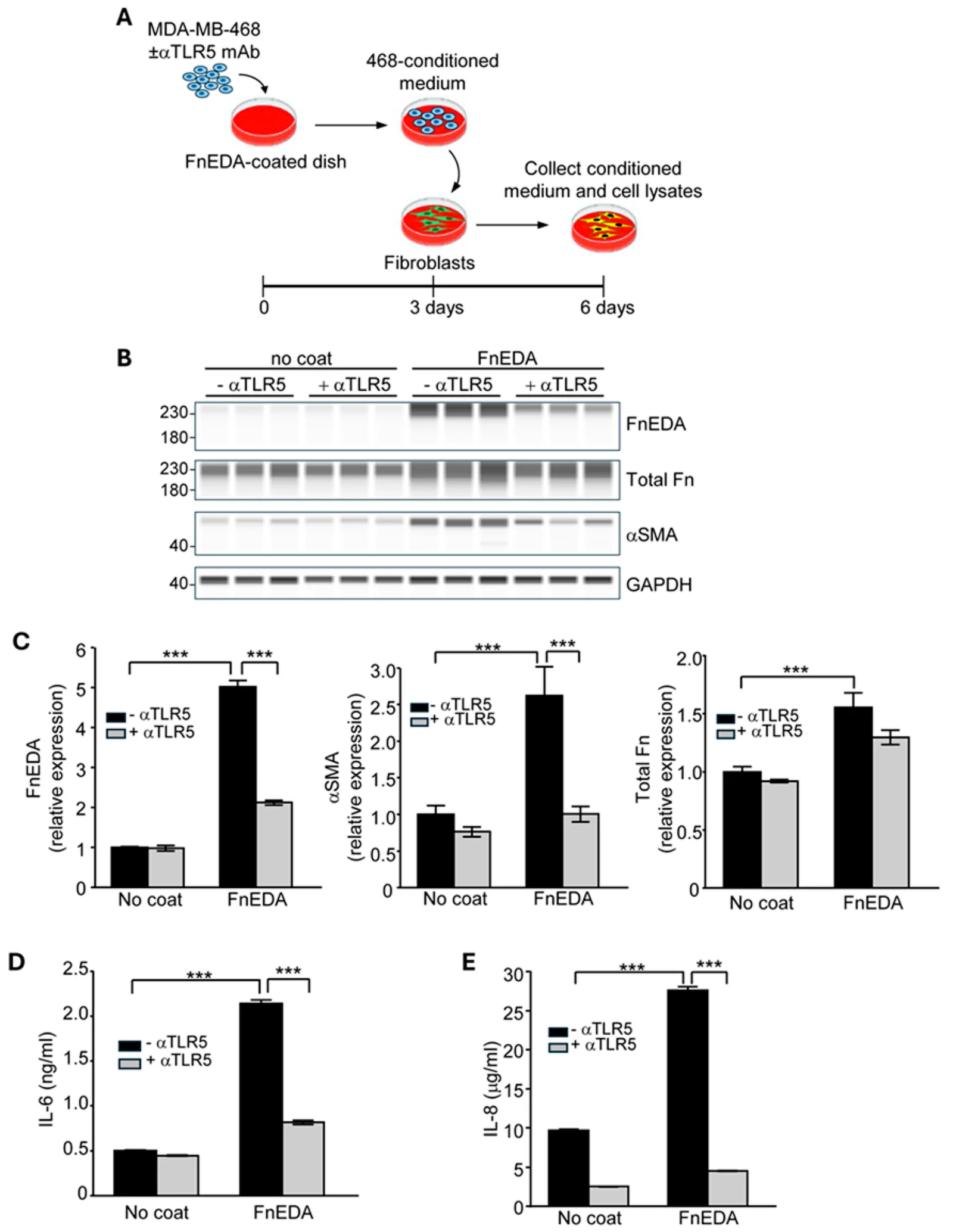
Fibronectin’s EDA domain induces MDA-MB-468 breast cancer cell secretion of profibrotic factors, which induce myofibroblast conversion in A1F fibroblasts. MDA-MB-468 breast cancer cells were seeded in complete medium in the presence or absence of 1 mg/mL anti-TLR5 neutralizing antibody (αTLR5) onto recombinant FnEDA-coated (10 mM in PBS) tissue culture dishes for 3 days (PBS-treated dishes used as control; no coat). The 468-conditioned medium was harvested, filter-sterilized to remove cells and debris, and then used to culture A1F fibroblasts for 3 days (**A**). A1F fibroblast-generated conditioned medium was harvested, and cells were lysed. Lysates were subjected to Simple-WES and probed for FnEDA, total Fn and αSMA (**B**). GAPDH served as loading control. Relative protein expression is shown in (**C**). Conditioned medium was analyzed by ELISA for IL-6 (**D**) and IL-8 (**E**). *** *p* < 0.001.

**Figure 6:**
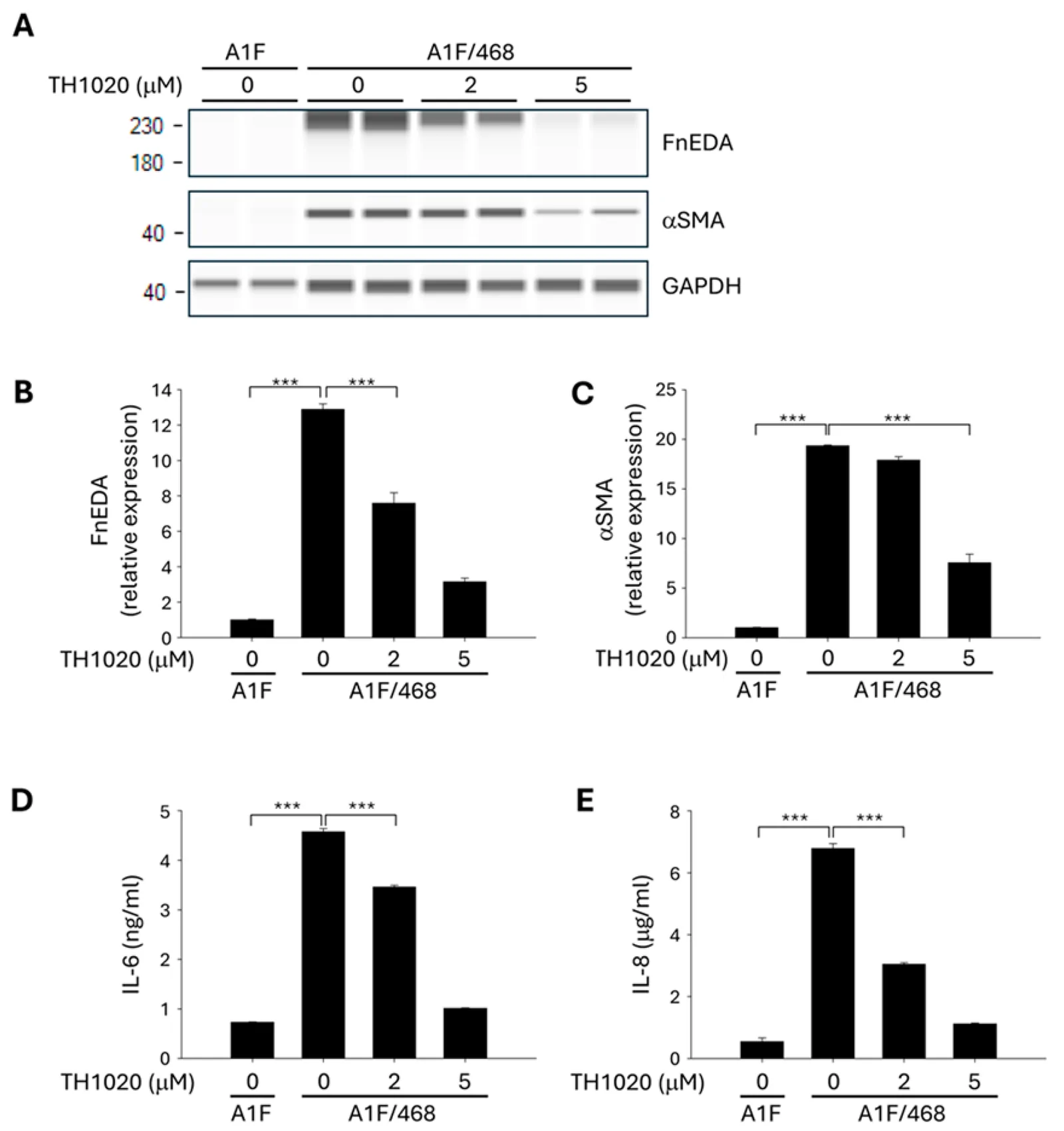
Inhibition of TLR5 in co-cultured A1F fibroblasts and MDA-MB-468 breast cancer cells suppresses expression of fibro-inflammatory markers. A1F fibroblasts were cultured alone (A1F, control) or in co-culture with MDA-MB-468 breast cancer cells (A1F/468) in complete medium and treated with increasing concentrations of the TLR5-selective inhibitor TH1020 for 3 days. Conditioned medium was harvested, and cell lysates prepared, analyzed using Simple-WES, and probed for FnEDA and αSMA (**A**). Relative protein expression levels were quantified in (**B**,**C**). The conditioned medium was assayed by ELISA for IL-6 (**D**) and IL-8 (**E**). *** *p* < 0.001.

Previous studies have shown that FnEDA functions as a Damage-Associated Molecular Pattern (DAMP) molecule activating Toll-Like Receptors (TLR) to induce expression of inflammatory genes in both immune and non-immune cells [25,26,27,28]. In the case of MDA-MB-468 cells, we have shown previously that the FnEDA domain activates TLR5, the prototype receptor for bacterial flagellin [13]. To evaluate whether the interaction of tumor cell TLR5 and fibroblast FnEDA is contributing to the conversion of A1F cells to CAFs, MDA-MB-468 cells were seeded onto FnEDA-coated plates and incubated for 72 h in the presence or absence of a blocking antibody to TLR5 (Figure 5A). Conditioned medium was then collected and applied to the A1F cells for 72 h. A1F cell lysates were then collected and analyzed by WES. As shown in Figure 5B, the addition of conditioned medium from the 468 cells, which were seeded onto FnEDA-coated plates, resulted in the increased expression of both FnEDA and αSMA in the A1F cells. This conversion of A1F cells to CAFs was inhibited when MDA-MB-468 cells were preincubated with a blocking antibody to TLR5. These data are quantified in Figure 5C. Consistent with these findings, IL-6 and IL-8 release was also dependent on TLR5 (Figure 5D,E). Similar results were seen when the mixed cultures were treated with the TLR5 inhibitor, TH-1020 [15]. As shown in Figure 6A, WES analysis showed a dose-dependent decrease in expression of FnEDA and αSMA when mixed cultures were treated with TH-1020. These data are quantified in Figure 6B,C. TH-1020 also inhibited the release of IL-6 and IL-8, as shown in Figure 6D,E.

**Figure 7:**
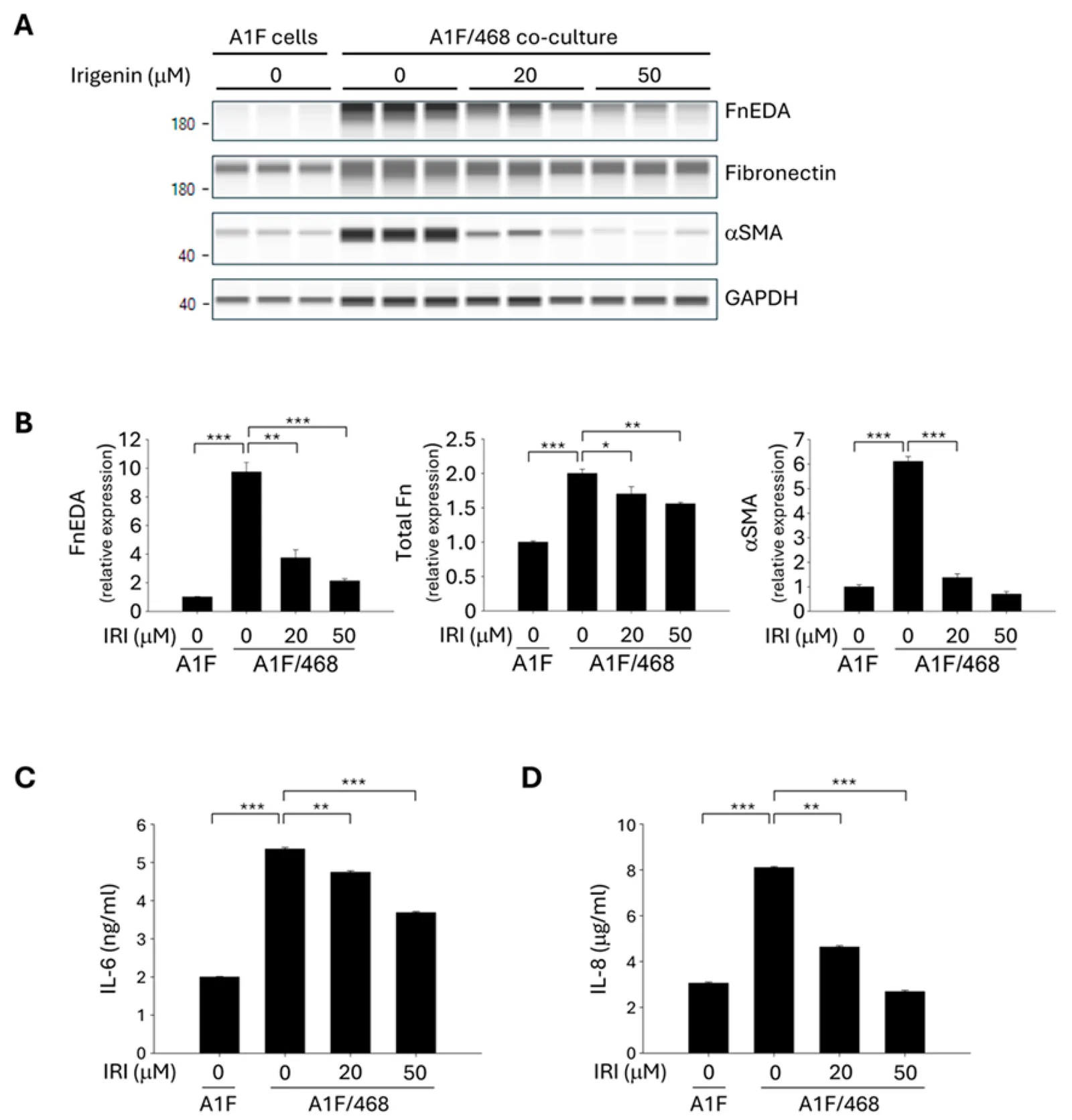
Blocking the EDA domain of fibronectin with Irigenin suppresses myofibroblast conversion in co-cultures of A1F fibroblasts and MDA-MB-468 breast cancer cells. A1F fibroblasts were cultured alone (as control) or in co-culture with MDA-MB-468 breast cancer cells (A1F/468) in complete medium and treated with increasing concentrations of irigenin (IRI), an inhibitor of integrin binding to the EDA domain of fibronectin, for 3 days. Cell lysates were analyzed using Simple-WES and probed for FnEDA, total Fn and αSMA (**A**) and relative expression levels quantified (**B**). GAPDH served as loading control. Conditioned medium was harvested and assayed by ELISA for IL-6 (**C**) and IL-8 (**D**). * *p* < 0.05; ** *p* < 0.01; *** *p* < 0.001.

As the EDA domain of fibronectin provides binding sites for the integrin-dependent activation of TGF-β as well as the initiation of TLR-dependent fibro-inflammation, we asked whether blocking the accessibility to fibronectin’s EDA domain could prevent the breast cancer cell-dependent conversion of fibroblasts to CAFs in our co-culture system. Irigenin is a flavonoid that binds to fibronectin’s EDA domain and has been demonstrated to have anti-inflammatory and anti-cancer properties [29,30,31]. As shown in Figure 7A, WES analysis indicated that the addition of Irigenin to the A1F/MDA-MB-468 co-cultures almost completely prevented the expression of both FnEDA and αSMA, with only a partial inhibition of total fibronectin. Quantification (Figure 7B) indicated that Irigenin completely inhibited the conversion of fibroblasts to CAFs. ELISA assays showed a partial inhibition of IL-6 expression in Irigenin-treated co-cultures (Figure 7C). In contrast, Irigenin completely blocked the increase in IL-8 expression seen in co-culture (Figure 7D).

**Figure 8:**
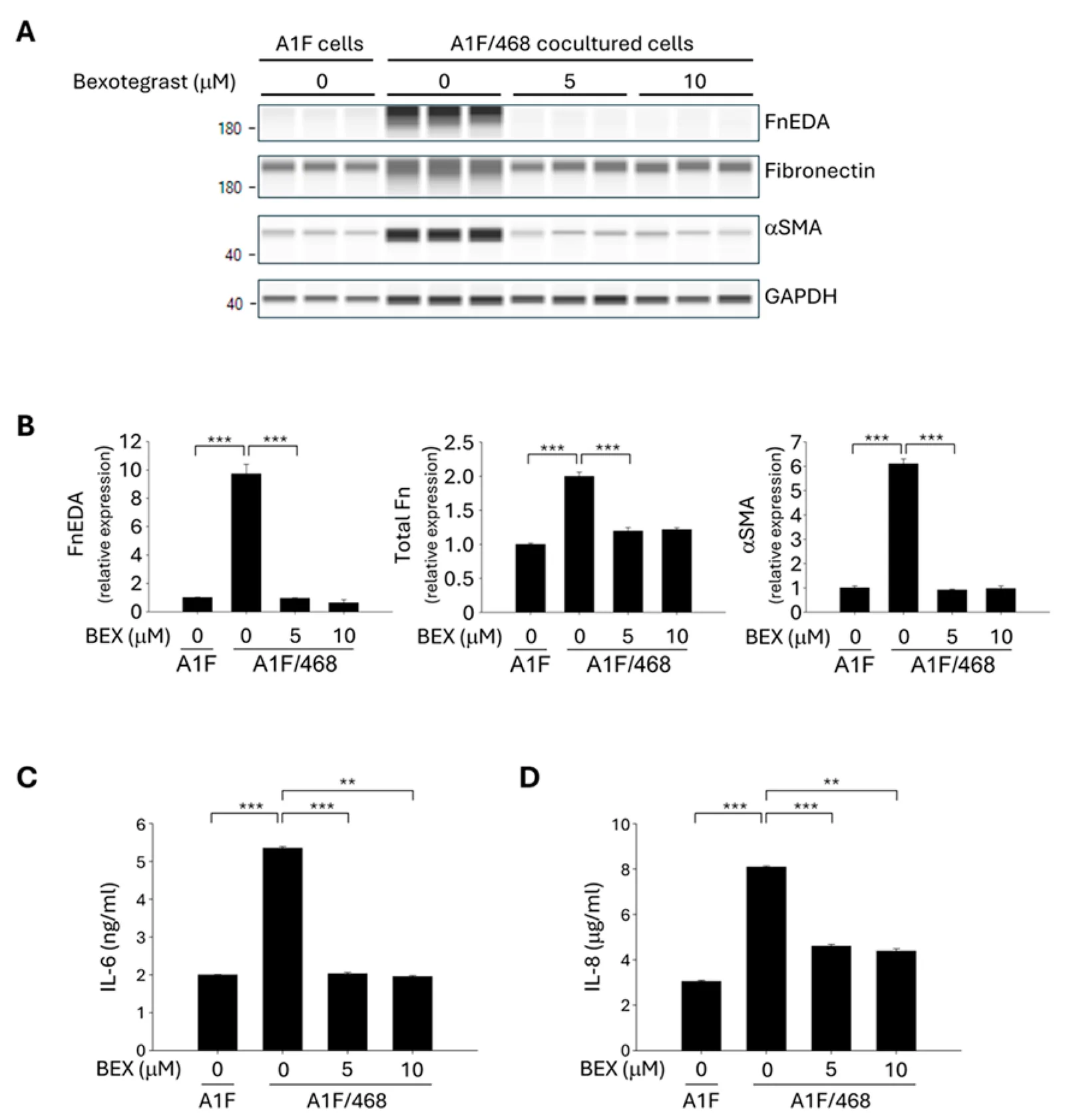
Selective inhibition of αv integrins involved in TGF-β activation suppresses A1F myofibroblast conversion in A1F/MDA-MB-468 co-cultures. A1F fibroblasts were cultured alone (A1F, as control) or in co-culture with MDA-MB-468 breast cancer cells (A1F/468) in complete medium and treated for 3 days with increasing concentrations of Bexotegrast (PLN-74809), an avβ6/αvβ1 integrin-selective inhibitor. Cell lysates were analyzed using Simple-WES and probed for FnEDA, total Fn and αSMA (**A**), and relative expression levels were quantified in (**B**). GAPDH served as loading control. Conditioned medium was harvested and assayed by ELISA for IL-6 (**C**) and IL-8 (**D**). ** *p* < 0.01; *** *p* < 0.001.

Activation of TGF-β bound to FnEDA depends on αvβ6 and αvβ1integrins. To evaluate their contribution to CAF differentiation in co-culture, the co-cultures were treated with Bexotegrast, a specific inhibitor of both αvβ6 and αvβ1 integrins [32,33]. MDA-MB-468 cells and A1F fibroblasts were placed in co-culture in complete medium with and without the addition of Bexotegrast. As shown in Figure 8A, in the absence of Bexotegrast, co-cultures showed increased expression of fibronectin, FnEDA and αSMA. Expression of these CAF markers was completely inhibited by the presence of Bexotegrast. Bexotegrast also inhibited the increased expression of fibronectin. These data are quantified in Figure 8B. As shown in Figure 8C,D, the increase in IL-6 and IL-8 was also prevented by Bexotegrast.

## 4. Discussion

Fibronectin matrix is upregulated in the stroma of human breast tumors and has been shown to promote cancer cell growth, migration, invasion, survival, and resistance to chemotherapy. The EDA isoform of fibronectin is considered a DAMP that binds TLRs [34,35] to activate innate immune responses in a variety of cell types [36,37]. Consequently, the molecular pathways controlling the tumor cell response to the fibronectin matrix are now regarded as potential drug targets for the management of chemo-resistant tumors [38]; however, the molecular pathways regulated by the pathological remodeling of the tissue fibronectin matrix within the stroma of solid tumors remain poorly characterized.

The significance of the EDA isoform of fibronectin to tumor progression was demonstrated in studies targeting splicing factors that control the inclusion of the EDA domain into fibronectin. These studies showed that elimination of the SRSF1 splicing factor prevented the formation of a stromal environment containing EDA fibronectin and completely prevented lung metastasis in a mouse model of breast cancer [7]. A second in vivo study was done to evaluate the role of the EDA isoform of fibronectin in the progression of open-angle glaucoma. Using transgenic mouse strains constitutively expressing only the EDA isoform of fibronectin or a second mouse strain expressing only fibronectin lacking the EDA domain, it was found that only the mice expressing the EDA isoform of fibronectin developed ocular hypertension, and this was dependent on TLR4 signaling [39]. These findings are consistent with our data showing that blocking of the EDA domain with Irigenin prevents myofibroblast conversion. The EDA domain is also the primary site of TGF-β activation as well as the binding site for Toll-like receptors. Together, these findings implicate only the EDA domain of fibronectin as the major driver of fibrotic disease. The EDA domain also provides a binding site for the latent form of TGF-β1, where it is subsequently activated by integrin receptors [40]. Bexotegrast blocks the binding of the αvβ1 and αvβ6 integrins to the latent form of TGF-β, thus preventing TGF-β activation. Bexotegrast has shown promise in clinical trials for the treatment of idiopathic pulmonary fibrosis, which results from a TGF-β-mediated increase in extracellular matrix deposition. It was found to be well tolerated, and patients showed decreases in collagen matrix and improvement in lung function [41]. Preclinical studies in mouse models have indicated that Bexotegrast may have some potential for the treatment of chronic kidney disease [42]. Consistent with this, a Phase II clinical study found that Bexotegrast was well tolerated and effective in treating primary sclerosing cholangitis and preventing subsequent fibrosis of the liver [43,44].

The tumor microenvironment also plays an essential but poorly understood role in the initiation, progression and treatment response of all solid tumors. The signaling networks between stromal and cancer cells are exceedingly complex and interdependent, occurring on a background of poorly understood mechanical signals that are activated in response to increasing tissue rigidity. Earlier studies have shown that the stiffened fibrotic microenvironment established by the CAFs also altered the metabolism of the cancer cells, thus promoting tumor progression [45]. Cellular interactions within the tumor microenvironment contribute to tumor progression. IL-6, IL-8, and TGF-β promote EMT, metastasis, immune suppression and resistance to therapy [46]. FnEDA activates TLR4 in stromal fibroblasts to initiate NF-κB activation and the subsequent release of pro-inflammatory cytokines, including IL-8 and IL-6, which are upregulated in many types of cancer, including breast cancer [47]. Previously, we have shown that FnEDA activates TLR4 in three different fibroblast cell lines: embryonic foreskin, adult dermal, and adult kidney. Although all three cell lines activated the NFκB pathway, the gene expression profiles were distinct [47]. There is also some specificity as to which TLRs regulate NFκB in triple negative breast cancer cells. In a previous study, we showed that MDA-MB-468 cell activation of NFκB-dependent cytokine release in response to FnEDA depends entirely on TLR5. Blocking antibodies to TLR2 and TLR4 had no effect. In contrast, both TLR2 and TLR5 regulate cytokine release in response to FnEDA in MDA-MB-231 cells, and their contribution to cytokine release is additive [13].

The fibronectin matrix serves as a scaffold for the binding of growth factors, other ECM molecules and cell surface receptors [48]. Fibronectin fibrils are mechanically sensitive and respond to increased contractile forces by unfolding Type III domains. This domain unfolding allows fibronectin fibers to stretch up to several times their length, thus altering the availability of various functional domains [49,50]. In previous studies, we have shown that mechanical unfolding of the Type III-1 domain of fibronectin also exposed an Fn DAMP, FnIII-1c, which activates the TLR4-dependent release of cytokines from fibroblasts [28,47,51]. Accumulating evidence now points to IL-8 playing a major role in the progression of breast cancer. Delineating the pathways that contribute to the control of prevailing IL-8 levels at various stages of breast cancer progression may provide novel targets for therapeutic intervention. IL-8 influences breast tumorigenesis by promoting invasivity [52], epithelial-mesenchymal transition (EMT) [53], mesenchymal and stem cell phenotypes [54,55,56,57], angiogenesis [58], chemoresistance and suppression of anti-tumor immunity [59]. The presence of IL-8 in breast tissues is negatively correlated with disease outcome and survival [60,61]. Previous findings have implicated the inflammatory cytokine IL-8 in the induction of breast cancer emergence from dormancy, indicating a role for this cytokine in the reemergence of actively growing cancer [62]. More recently, SB225002, an inhibitor of the IL8 receptor, CXCR2, was shown to reduce TNBC proliferation, invasion and metastasis in vitro and in vivo [20]. IL-8 can also play a role in chemo-resistance to paclitaxel and has been proposed as a possible drug target for paclitaxel-resistant TNBC [63]. We have shown previously that the TLR receptors regulating IL-8 release from TNBC cells in response to fibronectin DAMPs can be cell-type specific. In response to FnEDA, MDA-MB-468 cell activation of the NFκB signaling pathway and the secretion of IL-8 is entirely dependent on TLR5. However, MD-MB-468 cells did not activate NFκB or release cytokines in response to the fibronectin DAMP, FnIII-1c. In contrast, FnEDA stimulation of IL-8 from MDA-MB-231 TNBC cells depended on both TLR5 and TLR2, while cytokine release initiated by FN III-1c was exclusively regulated by TLR2 [13]. These findings suggest TLRs may provide an opportunity to develop targeted therapies for specific TNBC subtypes.

## 5. Conclusions

The data in this study are consistent with a model in which the TLR5 receptor on the MDA-MB-468 cells binds to EDA-fibronectin expressed by fibroblasts, thus inducing inflammatory cytokine expression. Expression of EDA fibronectin is regulated through TGF-β, whose synthesis is induced by TLR5 signaling in the tumor cells. The data are consistent with a model in which TLR5-mediated crosstalk between the cancer cells and the stromal fibroblasts creates a tissue microenvironment conducive to tumor growth and metastasis.

## Data Availability

The original contributions presented in this study are included in the article/Supplementary Material. Further inquiries can be directed to the corresponding author.

## Supplementary Materials

The following supporting information can be downloaded at: https://www.mdpi.com/article/10.3390/cancers18162693/s1.

## Abbreviations

EDA	Extra Domain A
TLR	Toll-like receptor
IL-8	Interleukin 8
IL-6	Interleukin 6
TNBC	Triple negative breast cancer
TGF-β	Transforming growth factor beta
CAF	Cancer-associated fibroblasts
α-SMA	alpha-smooth muscle actin
FnEDA	Fibronectin/extra domain A
DAMP	Damage-associated molecular pattern
NFκB	Nuclear factor kappa B
MAP	Mitogen-activated protein
DMEM	Dulbecco’s modified Eagle’s medium
FBS	Fetal bovine serum
ELISA	Enzyme-linked immunosorbent assay
FnIII	Fibronectin type III
EMT	Epithelial-mesenchmal transition
ECM	Extra cellular matrix
PBS	Phosphate-buffered saline
BSA	Bovine serum albumin
SEM	Standard error of the mean
