# Supplementary material for: Role of EDA Fibronectin and Toll-like Receptor 5 in the Development of the Tumor Microenvironment in Triple Negative Breast Cancer
Source: Cancers (Basel). 2026 Aug 20;18(16):2693. doi: 10.3390/cancers18162693 (PMC13510481; doi:10.3390/cancers18162693)
Supplement: Supplementary file 1 [file cancers-18-02693-s001.zip › cancers-4425389-supplementary.pdf]

Supplemental Figure S1

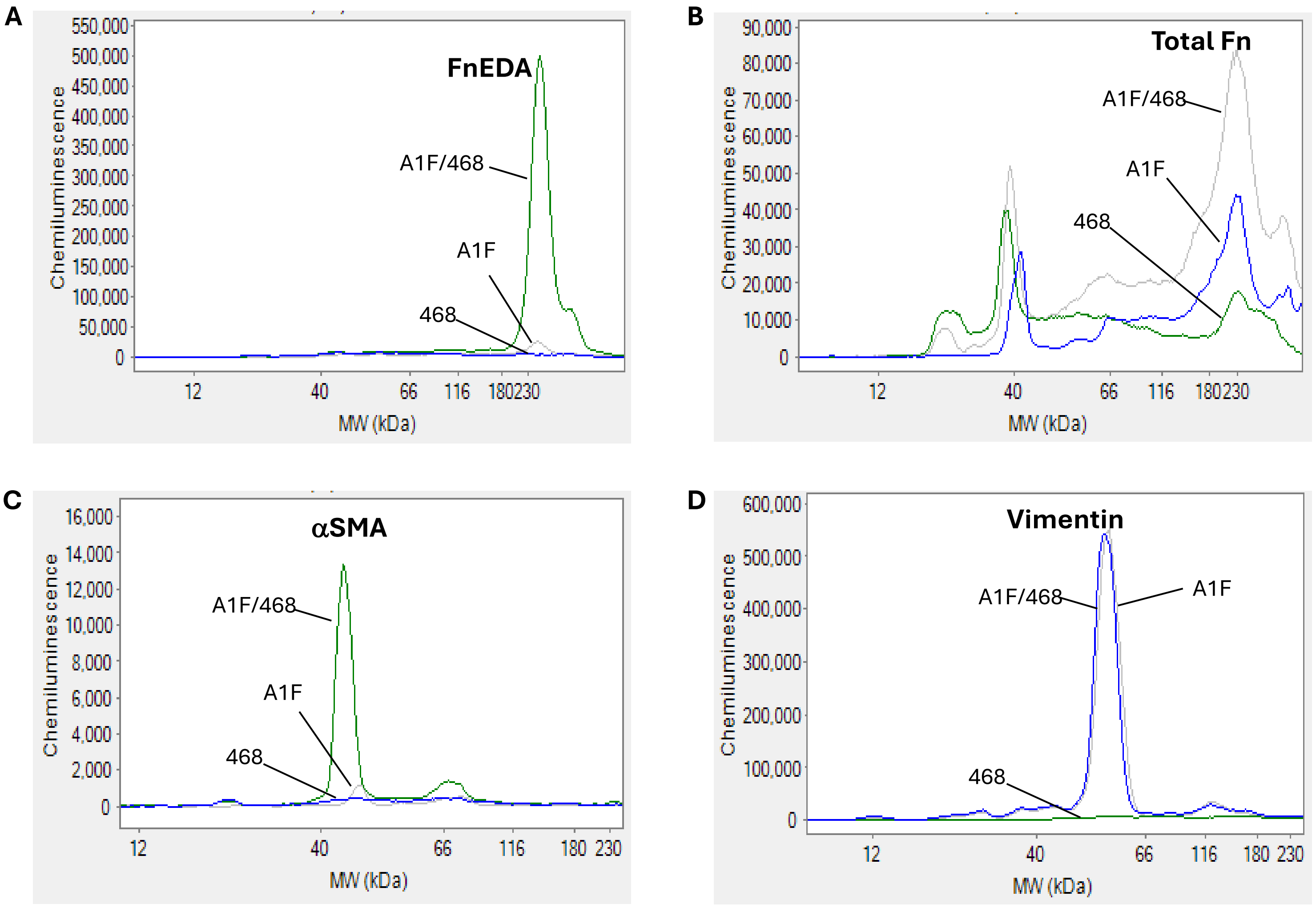

Figure S1. Electropherogram obtained from ProteinSimple Wes analysis of proteins separated based on their molecular weight where the peak area is proportional to the relative amount of that protein in the sample. Samples used are indicated and correspond to those described in the legend to Figure 2. Proteins detected and quantified include FnEDA (A), total Fn (B), αSMA (C), and vimentin (D).

Supplemental Figure S2

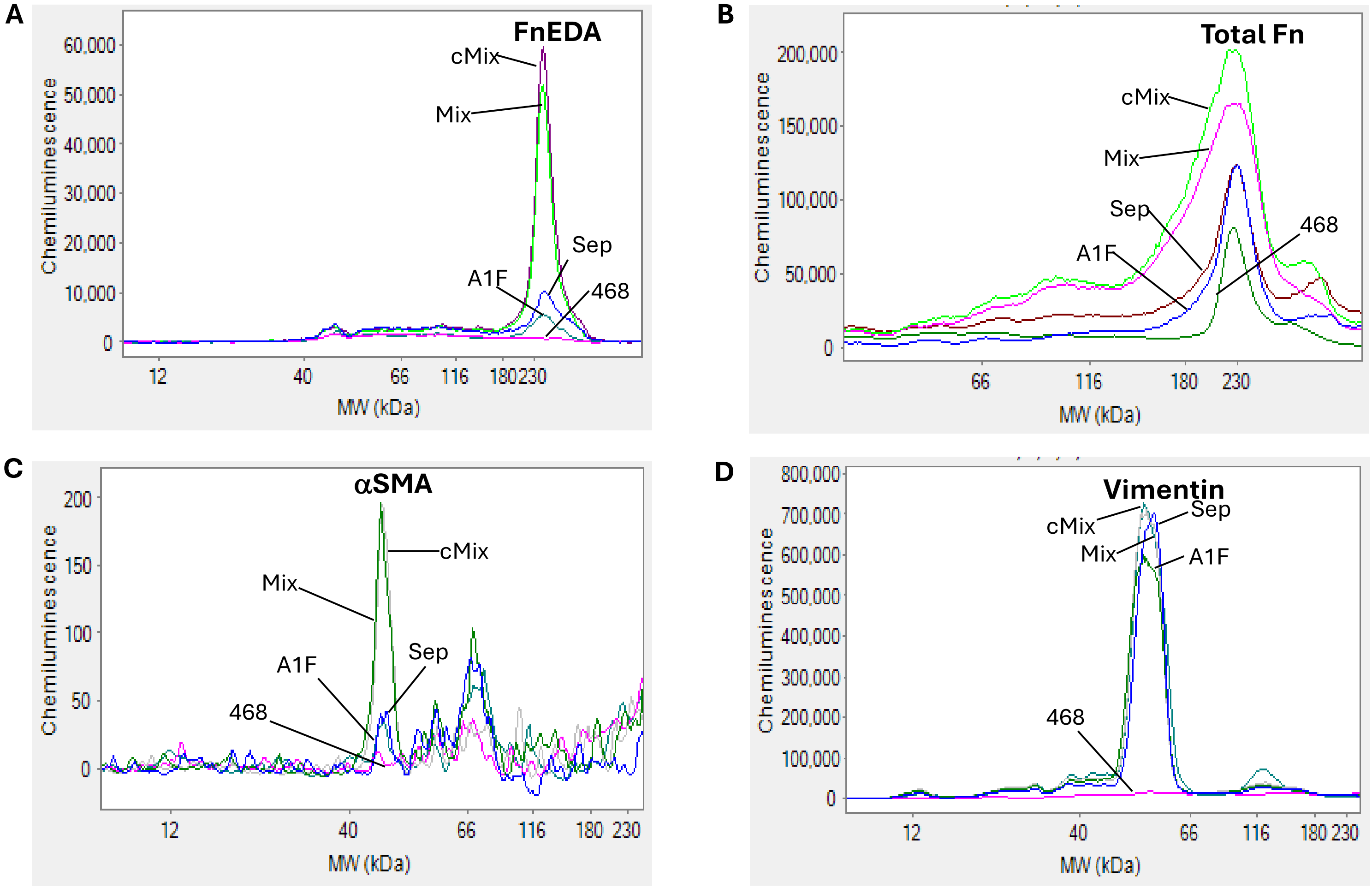

Figure S2. Electropherogram obtained from ProteinSimple Wes analysis of proteins separated based on their molecular weight where the peak area is proportional to the relative amount of that protein in the sample. Samples used are indicated and correspond to those described and quantified in the legend to Figure 3. Proteins detected include FnEDA (A), total Fn (B), αSMA (C), and vimentin (D).

Supplemental Figure S3

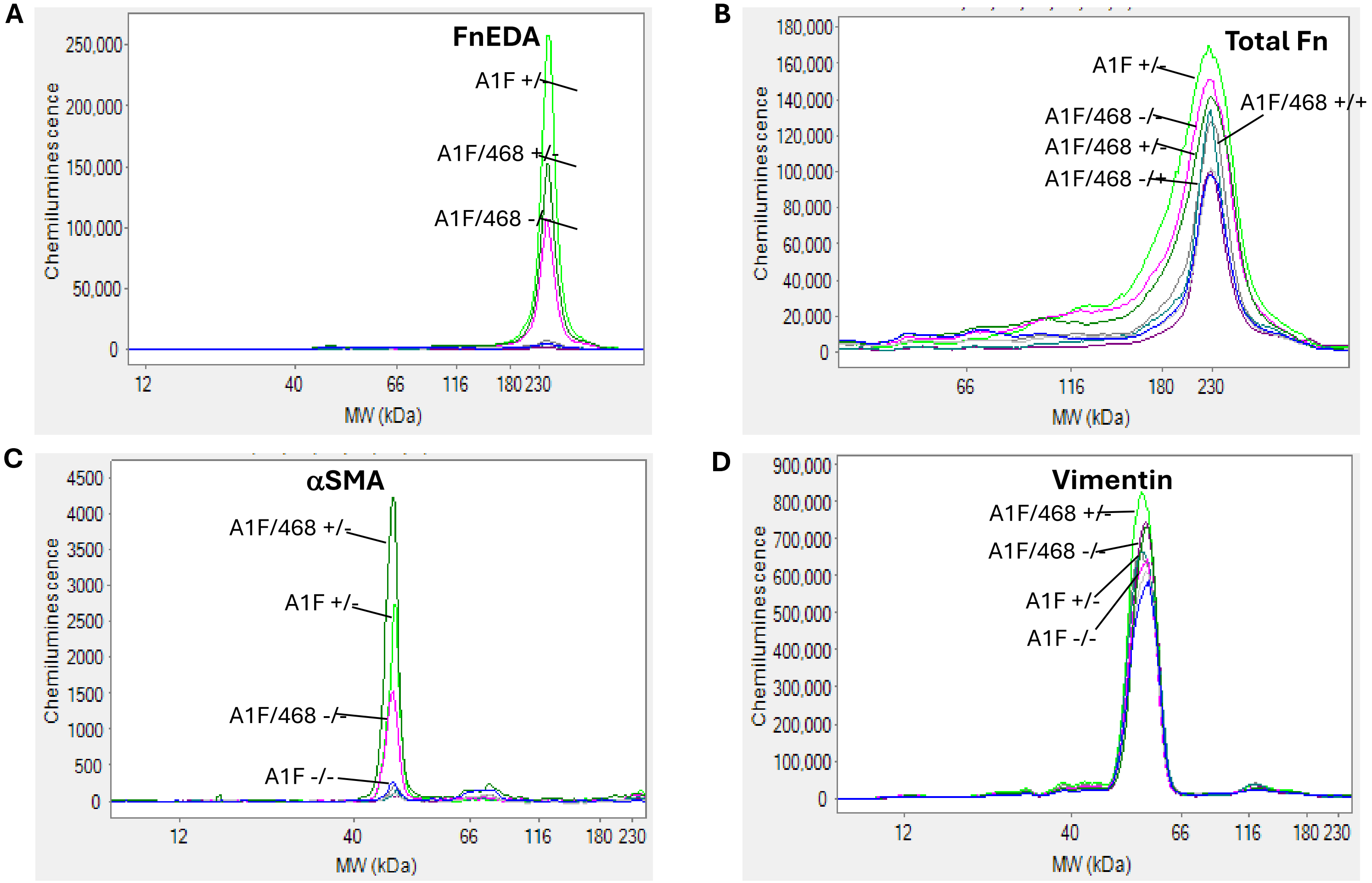

Figure S3. Electropherogram obtained from ProteinSimple Wes analysis of proteins separated based on their molecular weight where the peak area is proportional to the relative amount of that protein in the sample. Samples and treatments used are indicated and correspond to those described and quantified in the legend to Figure 4 (panel A). Proteins detected and quantified include FnEDA (A), total Fn (B), αSMA (C), and vimentin (D). -/-, no TGFβ or TGFβ inhibitor; +/-, TGFβ in absence of TGFβ inhibitor; +/+, both TGFβ and TGFβ inhibitor added; -/+, no TGFβ with added TGFβ inhibitor (for simplicity, some of the electropherograms of statistically non-significant treatments are not labeled in the figure).

Supplemental Figure S4

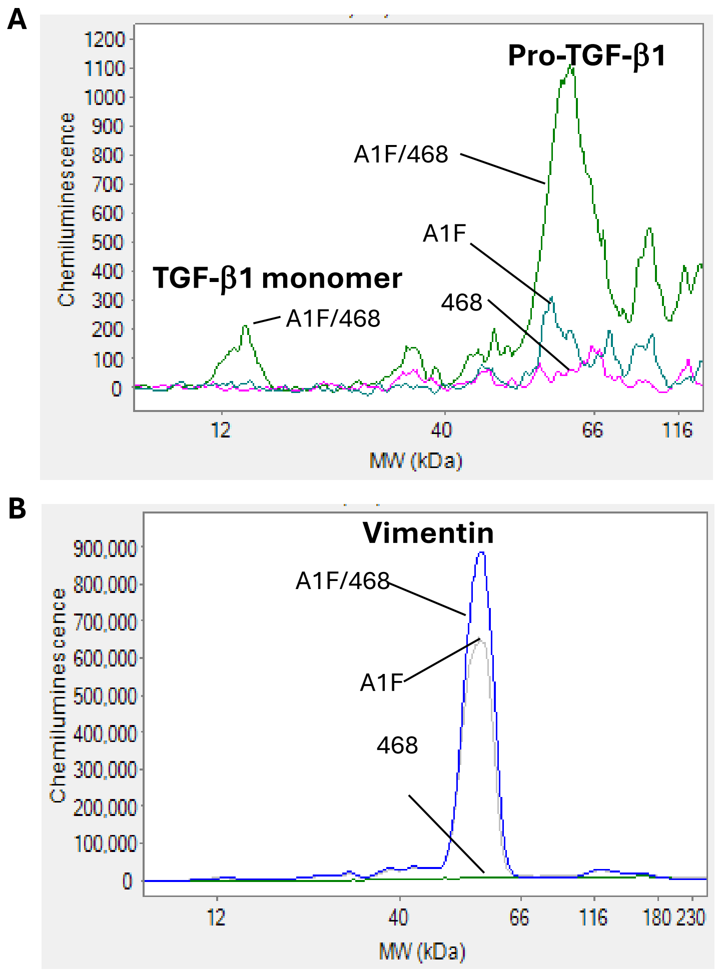

Figure S4. Electropherogram obtained from ProteinSimple Wes analysis of proteins separated based on their molecular weight where the peak area is proportional to the relative amount of that protein in the sample. Samples used are indicated and correspond to those described and quantified in the legend to Figure 4 (panel E). Proteins detected include pro-TGFβ and monomeric TGFβ (A) and vimentin (B).

Supplemental Figure S5

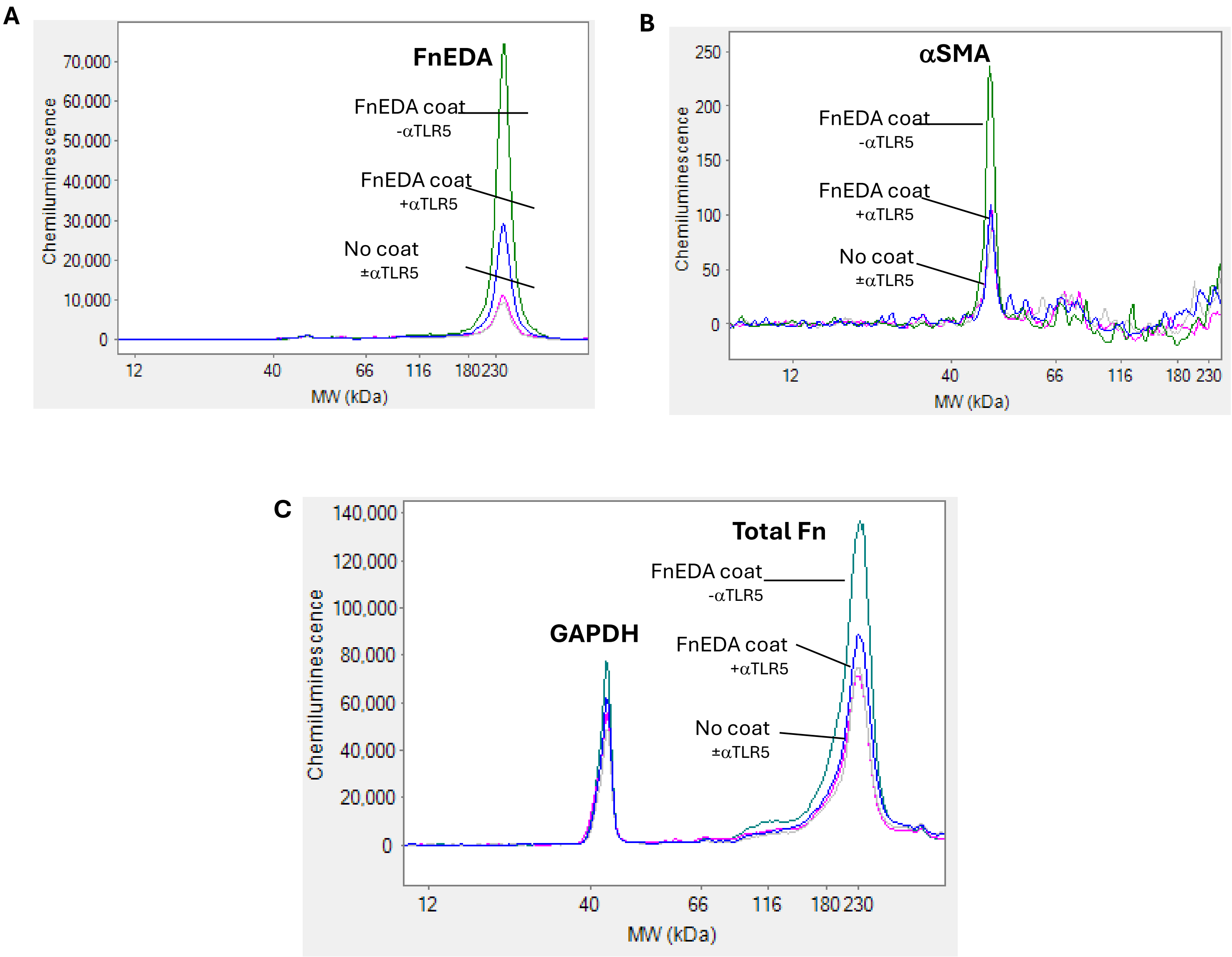

Figure S5. Electropherogram obtained from ProteinSimple Wes analysis of proteins separated based on their molecular weight where the peak area is proportional to the relative amount of that protein in the sample. Samples and treatments used are indicated and correspond to those described and quantified in the legend to Figure 5. Proteins detected include FnEDA (A), αSMA (B), and total Fn and GAPDH (C).

Supplemental Figure S6

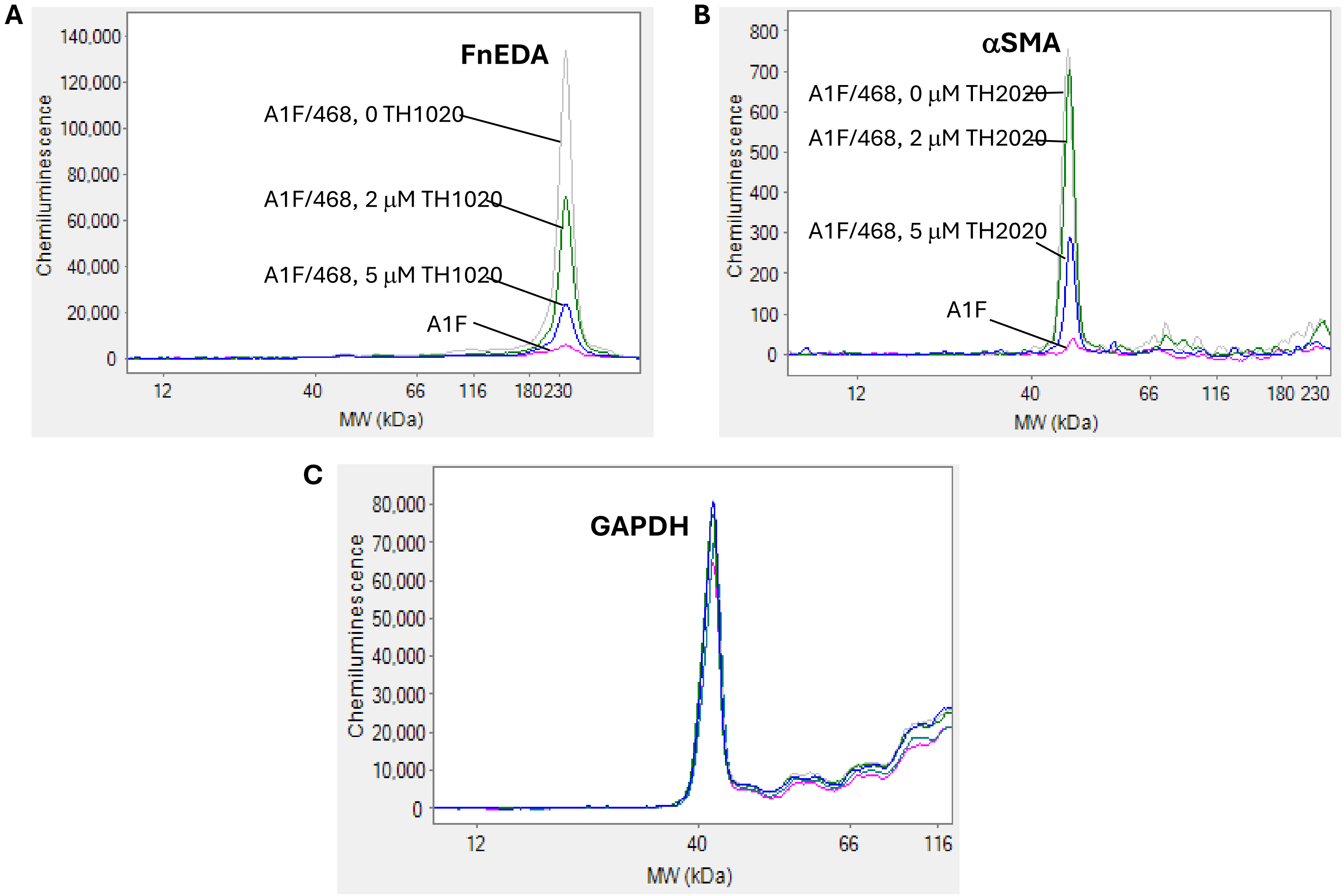

Figure S6. Electropherogram obtained from ProteinSimple Wes analysis of proteins separated based on their molecular weight where the peak area is proportional to the relative amount of that protein in the sample. TH1020 treated samples used are indicated and correspond to those described and quantified in the legend to Figure 6. Proteins detected include FnEDA (A), αSMA (B), and GAPDH (C).

Supplemental Figure S7

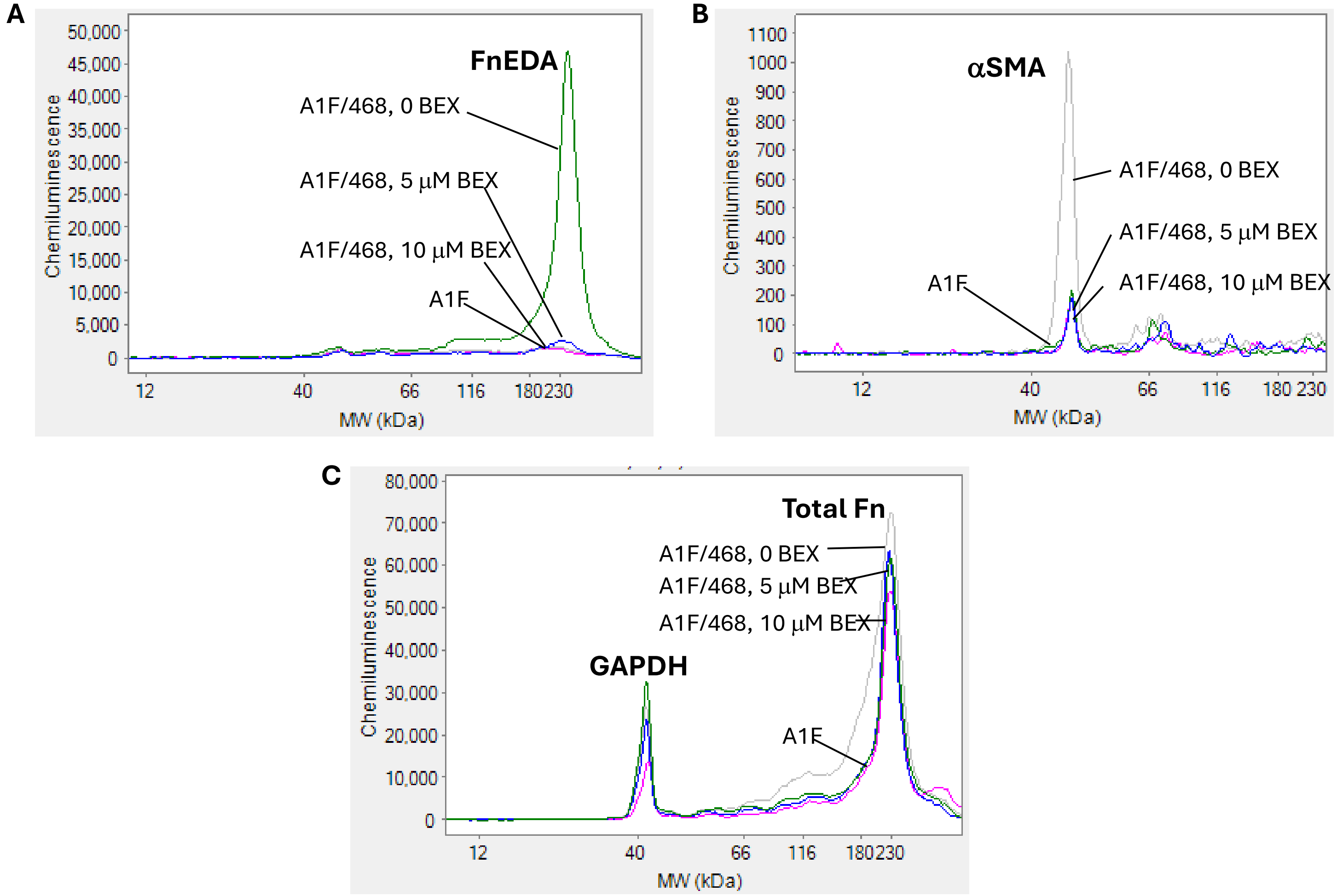

Figure S7. Electropherogram obtained from ProteinSimple Wes analysis of proteins separated based on their molecular weight where the peak area is proportional to the relative amount of that protein in the sample. Bexotegrast-treated samples (BEX) used are indicated and correspond to those described and quantified in the legend to Figure 7. Proteins detected include FnEDA (A), αSMA (B), and total Fn and GAPDH (C).

Supplemental Figure S8

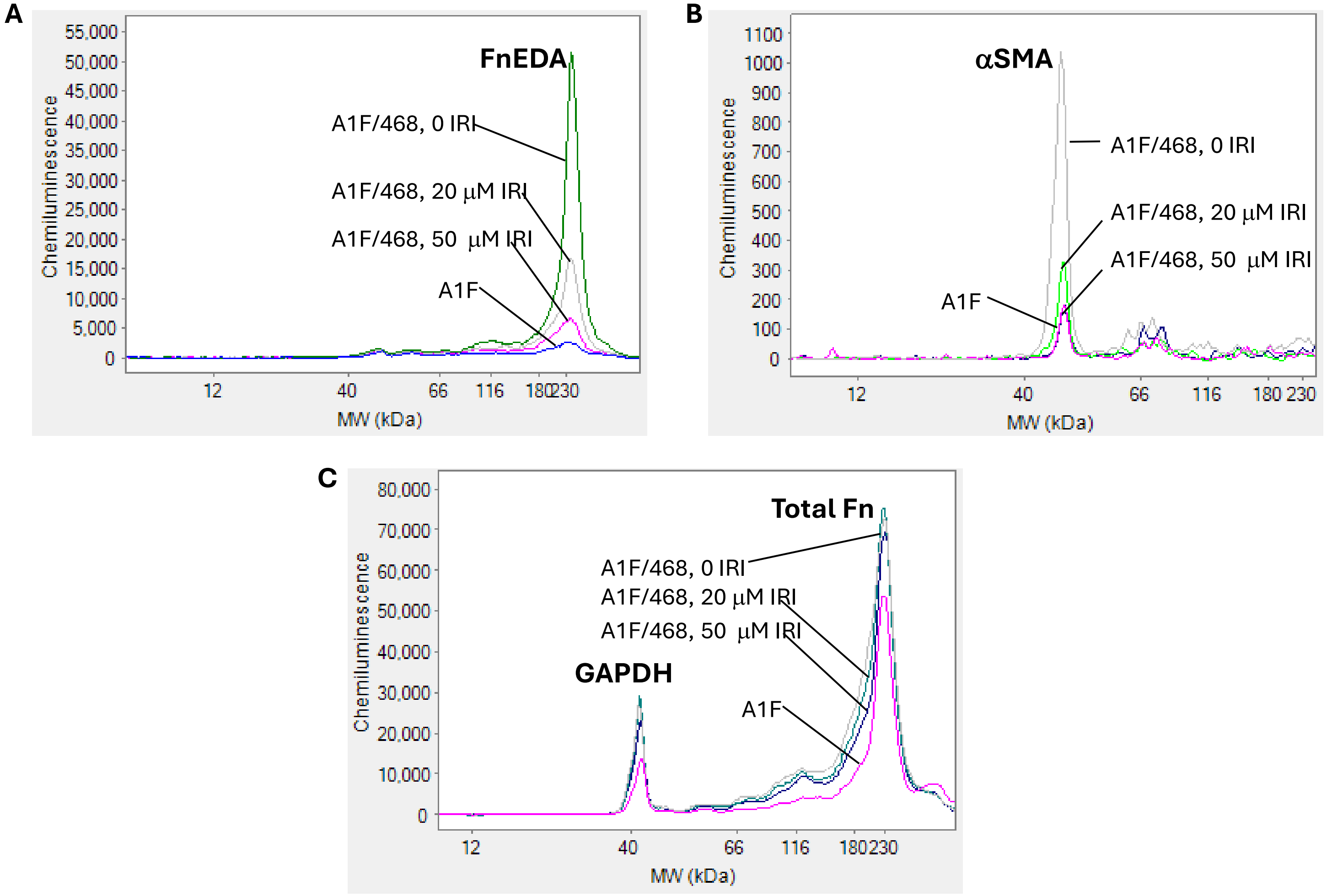

Figure S8. Electropherogram obtained from ProteinSimple Wes analysis of proteins separated based on their molecular weight where the peak area is proportional to the relative amount of that protein in the sample. Irigenin-treated samples (IRI) used are indicated and correspond to those described and quantified in the legend to Figure 8. Proteins detected include FnEDA (A), αSMA (B), and total Fn and GAPDH (C).
